# Migrasome-related LncRNA features predict immune microenvironment and prognosis in pancreatic cancer

**DOI:** 10.1038/s41598-025-24226-x

**Published:** 2025-11-18

**Authors:** Weihang Li, Yanrong Cao, Xin Sun, Ting Wang, Panling Xu, Ping Li

**Affiliations:** 1 Oncology Department of Integrated Traditional Chinese and Western Medicine, The First Affiliated Hospital of Anhui Medical University, Hefei, 230022 Anhui China; 2 Department of Integrated Traditional Chinese and Western Medicine, Anhui Medical University, Hefei, 230022 Anhui China

**Keywords:** PAAD, Migrasome-related LncRNA, Risk model, Immune microenvironment, Prognosis, Cancer, Computational biology and bioinformatics

## Abstract

The onset of pancreatic cancer is insidious, and the early symptoms are similar to those of common gastrointestinal diseases, which leads to easy neglect and misdiagnosis, which greatly affects the accuracy of survival prediction. Cell migration is the hallmark of malignant tumor and the key step of metastasis. Migrasome are involved in embryonic development, immune response, angiogenesis, inflammatory response, wound healing, and cancer metastasis in vivo. Considering the unknown association between migrasome and lncRNAs in pancreatic cancer, the purpose of this study was to identify migrasome-related lncRNAs (MRLs) and explore their prognostic value. In this study, we first analyzed the Pancreatic adenocarcinoma (PAAD) data in The Cancer Genome Atlas(TCGA) database and identified the correlation between MRLs and pancreatic cancer prognosis and immune infiltrating landscape. Secondly, four MRLs (MED14OS, AC141930.2, Z97832.2, LINC01091) were selected to construct a risk model as a prognostic feature. Kaplan-Meier survival analysis, Cox regression analysis, Nomogram and Time - dependent Receiver Operating Characteristic (ROC) Curve were then used to verify the accuracy of the model. And then, the Prognostic Risk Model were used in clinical to validate the accuracy. Finally, the correlation of immune score, tumor immune cell infiltration, tumor mutation load, tumor immune escape, and drug sensitivity of the risk model was systematically analyzed. The risk-prognosis model of MRLs was constructed. Survival analysis showed that the survival rate of high-risk subtypes was lower than that of low-risk subtypes. MRL features were an independent prognostic predictor, and the area under the subject working curve (AUC) for 1-year, 3-year, and 5-year were 0.667, 0.780, and 0.865, respectively. Prognosis MRLs is related to immune infiltrating landscape and can reflect the immune status, immune response, tumor mutation burden and drug sensitivity of pancreatic cancer patients. At the same time, this model can distinguish clinical patients well. The results of this study construct a predictive model of pancreatic cancer associated with migrasome, and clarify the relevance of this model to immunotherapy and so on. It provides a new idea for improving immunotherapy and drug therapy.

## Introduction

According to the 2023 Cancer Statistics Report^1^, the incidence of pancreatic cancer is still increasing, but among digestive cancers, pancreatic cancer has the worst overall survival rate at each stage. With the development of technology, although progress has been made in the detection and treatment of pancreatic cancer, the overall curative effect is not as good as expected^2^. Therefore, it is crucial to use appropriate prognostic models to predict the survival of patients and select appropriate treatment plans for patients, so as to improve the survival time of patients with pancreatic cancer.

Migrasome is a newly discovered organelle, which is a membranous structure formed at the tips or intersections of contracted filaments produced in the tail during cell migration^3,4^. Migrasome play an important role in cancer metastasis, either by releasing cell contents into the extracellular environment or by being absorbed by neighboring cells to transmit information^5^. Studies have confirmed^6^ that improper initiation or wrong direction of cell migration in the course of cancer can lead to greatly enhanced tumor aggressiveness or metastasis, resulting in invasive metastatic cancer. And the relationship between the migrasome and the tumor microenvironment is inseparable. Studies^7^ have shown that in the tumor microenvironment (TME), migrasome serve as chemotactic cues to enhance monocyte recruitment through feed-forward loops to promote angiogenesis. Given the important role of angiogenesis in tumor metastasis, these findings suggest that migrasome may play an important role in tumor progression. In addition, the migration-specific marker EOGT in hepatocellular carcinoma (HCC) is associated with TME immunoinfiltration in HCC, and its expression is significantly higher than in normal tissues, and is associated with advanced tumor stage and poor overall survival. Therefore, EOGT is considered to be an important indicator of poor prognosis in HCC patients^8^. Through detailed studies on the specific biomarkers of the migrasome^9,10^, it can be inferred that the migrasome is likely to affect these related TME by mediating the specific markers of the migrasome, thus affecting the progression of the tumor.

To predict the prognosis of pancreatic cancer, this study constructed a risk model based on four migrasome-related lncRNAs (MRLs). The risk model was systematically evaluated for its association with immune scores, immune cell infiltration, and other relevant features. This model may offer new insights into improving drug and immunotherapy responses for pancreatic cancer.

## Methods

### Data sources and processing

RNA sequencing data, mutation data, and clinical characteristics for pancreatic adenocarcinoma (PAAD) were obtained from The Cancer Genome Atlas (TCGA) database (https://portal.gdc.cancer.gov/)^11^. Patients with unavailable survival time were excluded from the analysis, resulting in a total of 185 samples. RNA expression data were translated using R software (version 4.4.1)^12^ and the Practical Extraction and Report Language (Perl, https://www.perl.org/about.html)^13^.

### Identification of prognostic migrasome-related LncRNAs

Using TCGA-PAAD as the expression dataset and a migrasome-related gene set as the reference database, co-expression analysis was conducted to identify migrasome-related lncRNAs.

Univariate Cox regression analysis was performed on these migrasome-related lncRNAs to identify those associated with prognosis. To eliminate false positives, Lasso-Cox regression analysis was used to refine the list of prognostic migrasome-related lncRNAs. For increased reliability and accuracy in prognostic predictions, multivariate Cox regression analysis was further employed to identify independent prognostic lncRNAs, excluding those that did not meet the criteria for being independent prognostic factors.

### Construction of the MRL prognostic risk model

A total of 185 samples were included in the study, excluding 7 normal tissue samples, leaving 178 patient samples for analysis. These samples were randomly split into two groups (1:1 ratio), with 89 samples in the training group and 89 in the testing group. The prognostic risk model based on migrasome-related lncRNAs was constructed using Lasso Cox regression analysis. Risk scores for each patient in both the training and testing groups were calculated based on the model’s formula. Patients were categorized into high- and low-risk subtypes based on the median risk score of the training group. Kaplan-Meier analysis was performed to compare overall survival (OS) and progression-free survival (PFS) between the high- and low-risk subtypes. Principal component analysis (PCA) was used to assess whether the model could distinguish between high- and low-risk subtypes.

### Validation of the MRL prognostic risk model

Cox regression analysis was performed to investigate the independence of the risk model using the “survival” R package. A nomogram was constructed using the “rms” R package to estimate the 1-year, 3-year, and 5-year survival rates. The prognostic performance of the risk model was further validated through time-dependent ROC analysis using the “time ROC” R package.

### Immune infiltration landscape

The immune scores of PAAD samples were calculated using the ESTIMATE R package. Immune cell infiltration and immune function were assessed via CIBERSORT, and immune cell composition was evaluated using the ssGSEA algorithm in the “GSVA” R package.

### Tumor mutational burden (TMB)

Mutation data for PAAD samples were obtained from the TCGA database in “maf” format. Mutation data were extracted from raw data using Perl scripts, and waterfall plots were generated using the “Maftools” R package.

### Immune response and drug sensitivity

The TIDE database was used to analyze tumor immune dysfunction and exclusion (TIDE). Drug sensitivity, represented as IC50 values, was obtained from the Cancer Drug Sensitivity Genomics (GDSC) database using the “oncoPredict” R package. IC50 values represent the drug concentration required to inhibit 50% of cell growth.

### Enrichment analysis

Differential analysis of all samples was performed using the LIMMA R package, identifying differentially expressed genes (DEGs). Genes with logFC > 0 and FDR < 0.05 were considered significantly different. Gene Ontology (GO) enrichment analysis (BP: biological process; CC: cellular component; MF: molecular function) and Kyoto Encyclopedia of Genes and Genomes (KEGG) pathway analysis were performed. Additionally, Gene Set Enrichment Analysis (GSEA) was used to explore differentially enriched KEGG pathways between the high- and low-risk subgroups.

### Clinical validation of MRLs prognostic risk model

In order to verify the accuracy of MRLs prognostic risk model in predicting the PFS and OS of pancreatic cancer patients, we collected the biopsy tissue samples and clinical OS information of patients with newly diagnosed advanced pancreatic cancer in the Cancer Center of Integrated Traditional Chinese and Western Medicine of the First Affiliated Hospital of Anhui Medical University, and approval was obtained from the ethics committee (NO. PJ2024-05–74).The cancer tissue of the patients were frozen and milled, and then Trizol (Beyotime, China) was used to extract total RNA. After the RNA-seq library was constructed, the total RNA was sent for RNA sequencing (Shanghai Oebbiotech Co, LTD, China). Substitute the expression levels of the four MRLs of each patient into the model calculation formula to calculate the risk score of each patient. Then, based on the median risk score of the training group, these patients were divided into high - risk and low - risk groups. KM survival curve was used to determine whether there was a difference in PFS and OS between patients with high and low risk.

## Results

### Identification of prognostic migrasome-related LncRNAs

**Fig. 1:**
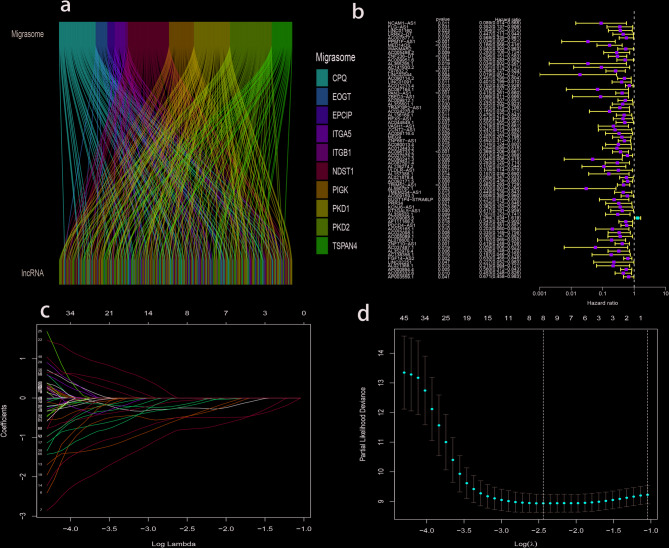
Identification of Prognostic Migrasome-Related lncRNAs. (**a**) Sankey diagram showing the correlation between migrasome genes and lncRNAs. (**b**) Univariate Cox regression analyses suggest that MRLs were significantly correlated with the overall survival (OS) of patients with PAAD. (**c**,** d**) LASSO analyses showing the minimum lambda and coefficient of the prognostic MRLs.

185 samples of RNA sequencing data, clinical follow-up data and tumor mutation from TCGA database, 11 migrasome related genes from genecards database (https://www.genecards.org/) and three paper^9,14,15^ include: ITGB1, ITGA5, EOGT, CPQ, PIGK, NDST1, TSPAN4, EPCIP, PKD2, PKD1, TMX2-CTNND1. LncRNAs associated with migrasomes were identified via co-expression analysis (Fig. 1a). In order to explore the prognostic value of migrasome-related lncRNAs in PAAD patients, univariate Cox regression analysis was performed, and prognostic related lncRNAs were selected according to *P* < 0.05 (Fig. 1b). With that, lncRNAs associated with false positive prognosis were eliminated by Lasso analysis, and eight migrasome lncRNAs were obtained (Fig. 1c, d), including MED14OS, AC141930.2, Z97832.2, LINC01091, NCAM1-AS1, PPM1F-AS1, RFX5-AS1, and MEG9.

### Establishment of the risk model using MRL prognostic features

**Fig. 2:**
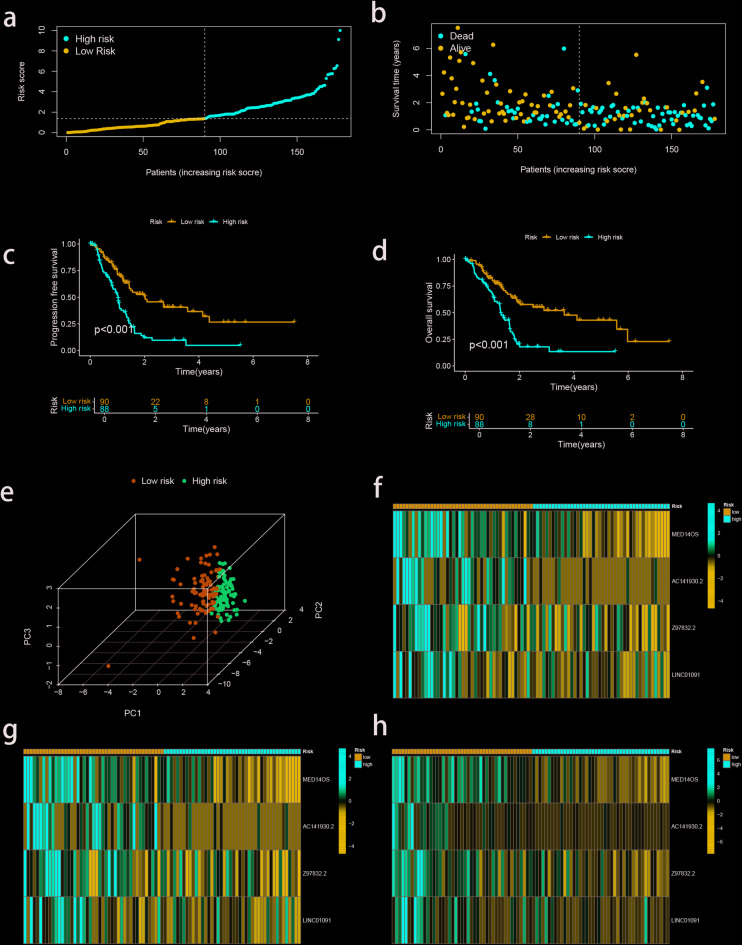
Establishment of the Risk Model. (**a**,** b**) Distribution of risk score and scatter dot plot showing the correlation of survival time and risk score. (**c**) Kaplan-Meier survival curve suggests that the OS of patients in the high-risk group is significantly shorter than that in the low-risk group. (**d**) Kaplan-Meier survival curve suggests that the PFS of patients in the high-risk group is significantly shorter than that in the low-risk group. (**e**) PCA illustrates a significant difference between the low-risk group and high-risk group based on the MRL prognostic signature. (**f**) Heatmap of all patients. (**g**) Heatmap of test. (**h**) Heatmap of train.

Multivariate Cox regression analysis was used to evaluate the effects of these eight prognostic migrasome-related lncRNAs on OS and clinical outcomes in PAAD patients. Finally, four MRLs, including MED14OS, AC141930.2, Z97832.2 and LINC01091, were identified to establish a prognostic risk model. Lasso Cox regression analysis was used to construct prognostic risk models from 4 selected MRLs that were identified as protective factors. The risk scoring formula is as follows: RiskScore = MED14OS* (−1.52801576009255) + AC141930.2* (−2.6087818589119) + Z97832.2* (−0.85431223759962) + LINC01091* (−0.729627953975936). PAAD patients were divided 1:1 into training (*n* = 89) and validation (*n* = 89) groups, with no clinical trait differences between the groups (Table 1). Patients were classified into high-risk and low-risk subtypes according to the median risk score of the training group. The scatter plot shows a negative correlation between risk score and survival time (Fig. 2a, b). Survival analysis of all patients showed that the OS and PFS of high-risk subtypes were shorter than those of low-risk subtypes (Fig. 2c, d). PCA revealed differences between high-risk and low-risk subtypes (Fig. 2e). In addition, heat map visualization showed that the expression of four MRLs was significantly increased in low-risk subtypes (Fig. 2f, g, h).

**Table 1:** Baseline characteristics of patients in this study.

Covariates	Type	Total	Test	Train	*P* value
Age	<=65	94(52.81%)	48(53.93%)	46(51.69%)	**0.8807**
	> 65	84(47.19%)	41(46.07%)	43(48.31%)	
Gender	Female	80(44.94%)	39(43.82%)	41(46.07%)	**0.8802**
	Male	98(55.06%)	50(56.18%)	48(53.93%)	
Grade	G1	31(17.42%)	15(16.85%)	16(17.98%)	**0.7957**
	G2	95(53.37%)	50(56.18%)	45(50.56%)	
	G3	48(26.97%)	21(23.6%)	27(30.34%)	
	G4	2(1.12%)	1(1.12%)	1(1.12%)	
	Unknow	2(1.12%)	2(2.25%)	0(0%)	
Stage	Stage I	21(11.8%)	13(14.61%)	8(8.99%)	**0.2415**
	Stage II	147(82.58%)	73(82.02%)	74(83.15%)	
	Stage III	3(1.69%)	0(0%)	3(3.37%)	
	Stage IV	4(2.25%)	2(2.25%)	2(2.25%)	
	Unknow	3(1.69%)	1(1.12%)	2(2.25%)	
T	T1	7(3.93%)	3(3.37%)	4(4.49%)	**0.2828**
	T2	24(13.48%)	14(15.73%)	10(11.24%)	
	T3	142(79.78%)	71(79.78%)	71(79.78%)	
	T4	3(1.69%)	0(0%)	3(3.37%)	
	Unknow	2(1.12%)	1(1.12%)	1(1.12%)	
M	M0	80(44.94%)	43(48.31%)	37(41.57%)	**1**
	M1	4(2.25%)	2(2.25%)	2(2.25%)	
	Unknow	94(52.81%)	44(49.44%)	50(56.18%)	
N	N0	49(27.53%)	28(31.46%)	21(23.6%)	**0.4391**
	N1	124(69.66%)	61(68.54%)	63(70.79%)	
	Unknow	5(2.81%)	0(0%)	5(5.62%)	

### Performance of the risk model in the training and validation cohorts

**Fig. 3:**
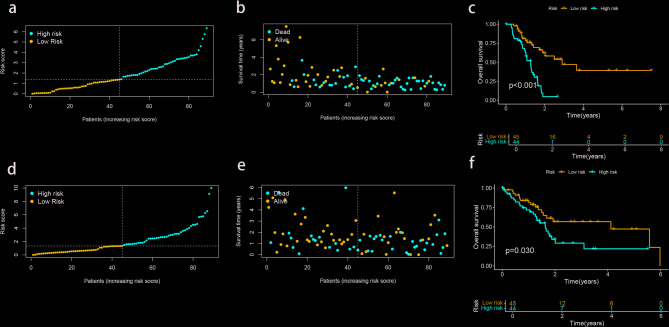
Construction of the risk model in the training and validation cohorts. (**a**,** b**) The distribution of risk score and scatter dot plot shows the correlation of risk score and OS in the training cohort. (**c**) Kaplan-Meier survival curve suggests that the OS of patients with high-risk score is significantly lower than those with low-risk score in the training cohort. (**d**,** e**) The distribution of risk scores and scatter dot plot of patients with PAAD based on the MRL prognostic signature in the validation cohort. (**f**) Kaplan-Meier survival curve suggests that the OS of patients with high-risk score is significantly lower than those with low-risk score in the validation cohort.

To confirm the effect of this model, PAAD patients were divided into a training group (*n* = 89) and a validation group (*n* = 89). In the training group, scatter plots showed a negative correlation between survival time and risk scores (Fig. 3a, b). Kaplan-Meier analysis showed that OS of high-risk subtypes was significantly lower than that of low-risk subtypes (*P* < 0.001 Fig. 3c). Scatter plots and Kaplan-Meier analyses observed similar trends in the validation group (Fig. 3d, e, f), with significantly lower OS rates for the high-risk subtype compared to the low-risk subtype (*P* = 0.030, Fig. 3f).

### MRL model features as independent prognostic factors

**Fig. 4:**
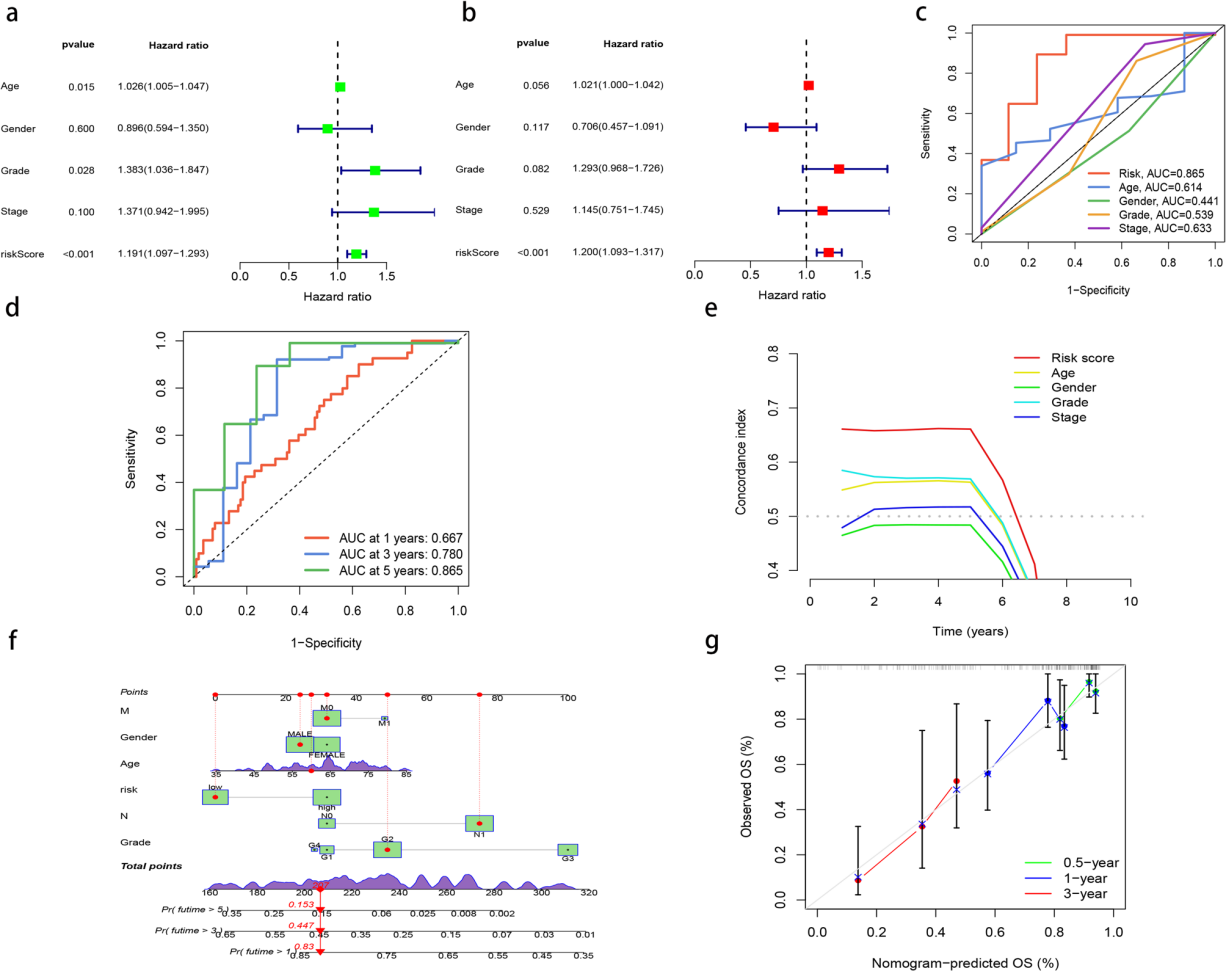
Independent prognostic analysis of MRLs. (**a**) Univariate Cox regression analysis. (b) Multivariate Cox regression analysis. (**c**) The predictive performance of the model is superior to that of other clinical factors. (**d**) The 1-year, 3-year, and 5-year ROC. (**e**) Curve of C-index. (**f**) Nomogram for evaluating the prognosis of patients with PAAD. (**g**) calibration curve of the nomogram: Solid lines close to diagonal dashed lines indicate better predictions.

Cox regression analysis was performed to verify whether the MRL prognostic model was an independent prognostic factor for PAAD. Univariate Cox regression analysis showed that Age (HR = 1.026, *P* = 0.015), Grade (HR = 1.383, *P* = 0.028), and riskScore (HR = 1.191, *P* < 0.001) were the prognostic factors associated with PAAD (Fig. 4a). Multivariate Cox regression analysis showed that riskScore(HR = 1.200,*P* < 0.001) was an independent predictor of PAAD prognosis (Fig. 4b), which confirmed the prognostic ability of MRLs features of migrasome. ROC results showed that the AUC of riskScore was 0.865 (Fig. 4c). The 1-year, 3-year and 5-year AUC were 0.667, 0.780, and 0.865, respectively (Fig. 4d). ROC and C-index results showed that riskScore had better prognostic ability than other clinical factors (Fig. 4c, e). We also constructed a new nomogram model integrating risk scores and clinicopathological features (Fig. 4f). Since the survival period of pancreatic cancer is relatively short, the calibration curve only includes 0.5 - year, 1 - year, and 3 - year time points. The calibration curve of the nomogram shows a high degree of consistency between the predicted and observed survival probabilities (Fig. 4g). These data jointly explain the accuracy of the risk model of four selected MRLs for the prognosis of PAAD.

### Subgroup analysis of the MRL risk model based on clinical features

**Fig. 5:**
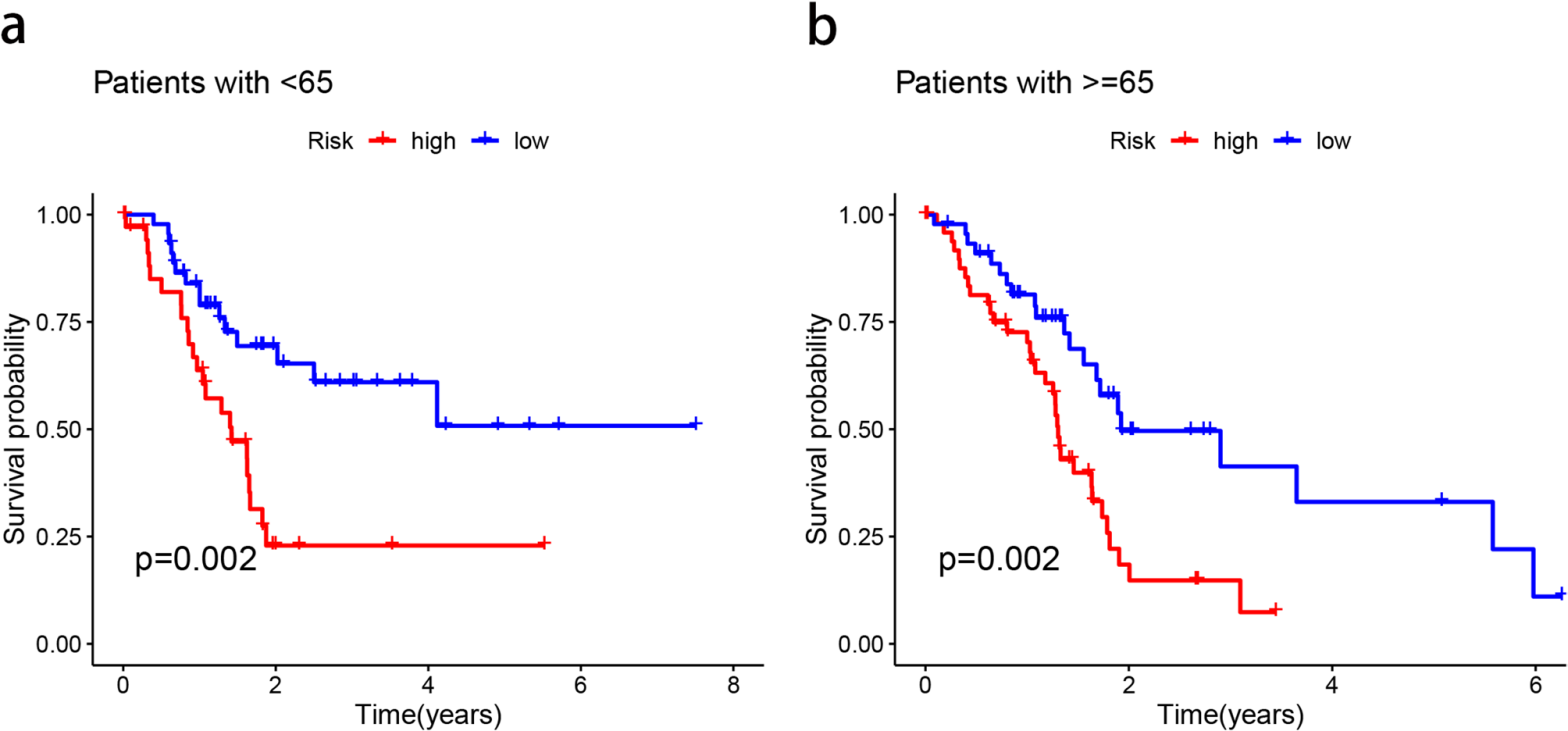
Subgroup Kaplan–Meier survival curve. Kaplan**–**Meier survival curve analysis shows the OS rates of low- and high-risk patients with PAAD stratified by (**a**,** b**) age < 65 vs. ≥ 65.

We grouped the samples according to whether the age of the sample was greater than or equal to 65 years old: ≥65 years old; <65 years old, and then performed Kaplan-Meier survival analysis for the two groups separately, and found that the MRL risk model not only produces accurate prediction of the survival rate of the patients with lower age (< 65 years old), but also applies to the patients with higher age (≥ 65 years old). It was equally applicable (Fig. 5a, b).

### Correlation of the risk model with pancreatic cancer immune scores and immune cell infiltration (ICI)

**Fig. 6:**
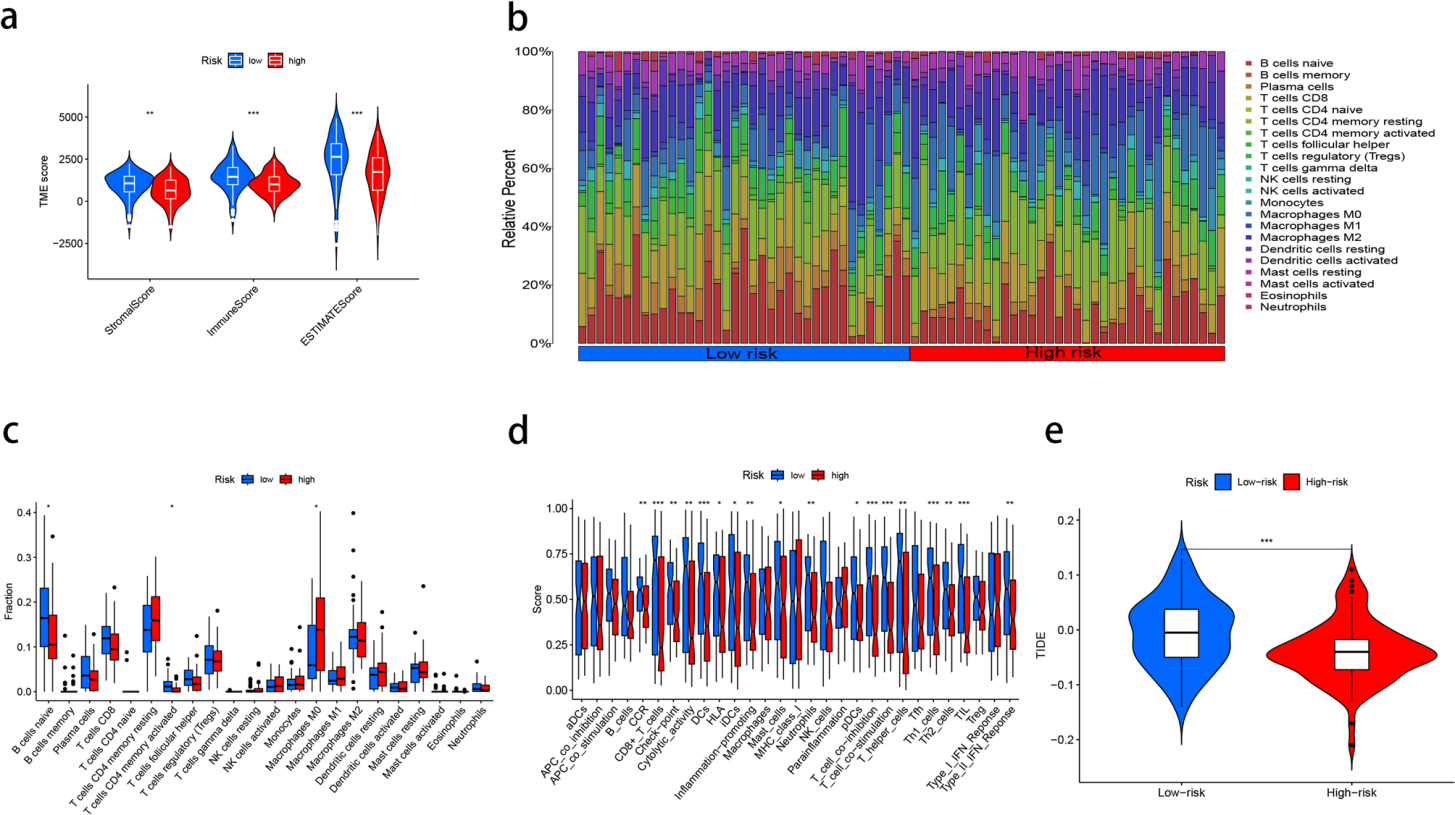
Immune Scores and Immune Cell Infiltration. (**a**) Immune Score of PAAD. (**b**,** c**,** d**) The fraction of immune cells calculated by CIBERSORT and ssGSEA algorithms. (**e**) Tumor immune dysfunction and exclusion (TIDE) analysis.

The association between immune scores and risk scores was investigated firstly. The tumor stroma score (*P* < 0.01), immune score (*P* < 0.001) and total score (*P* < 0.001) in high-risk group were significantly lower than those in low-risk group (Fig. 6a). This means that the tumor tissue in the low-risk group has a higher proportion of tumor interstitial components and more immune cell infiltration than that in the high-risk group. Subsequently, CIBERSORT formula and ssGSEA were used to evaluate the differences in immune cell infiltration level and immune function between high-risk and low-risk groups (Fig. 6b, c, d). CIBERSORT results showed: Patients with high-risk subtypes had higher levels of M0 macrophages (*P* < 0.05), while the infiltration levels of naive B cells (*P* < 0.05) and activated memory CD4 + T cells (*P* < 0.05) were lower than those of patients with low-risk subtypes. The results of ssGSEA showed that chemokine activity (*P* < 0.01), checkpoint activity (*P* < 0.01), tumor-infiltrating lymphocyte activity (*P* < 0.001), mast cell activity (*P* < 0.05), CD + 8 T cell activity (*P* < 0.001), helper T cell activity (*P* < 0.001) in low-risk subtypes were all the same higher than patients with high-risk subtypes. In addition, to predict immune status and potential immune checkpoint blocking (ICB) response, the correlation of prognostic models with immune checkpoint expression and ICB response was investigated based on the TCGA dataset. The potential ICB response was predicted using the Tumor Immune Dysfunction and Exclusion (TIDE) algorithm, which showed that the low-risk group had more potential ICB response than the high-risk group, and the high-risk group showed a more favorable response to immunotherapy (Fig. 6e).

### Tumor mutational burden (TMB) analysis

**Fig. 7:**
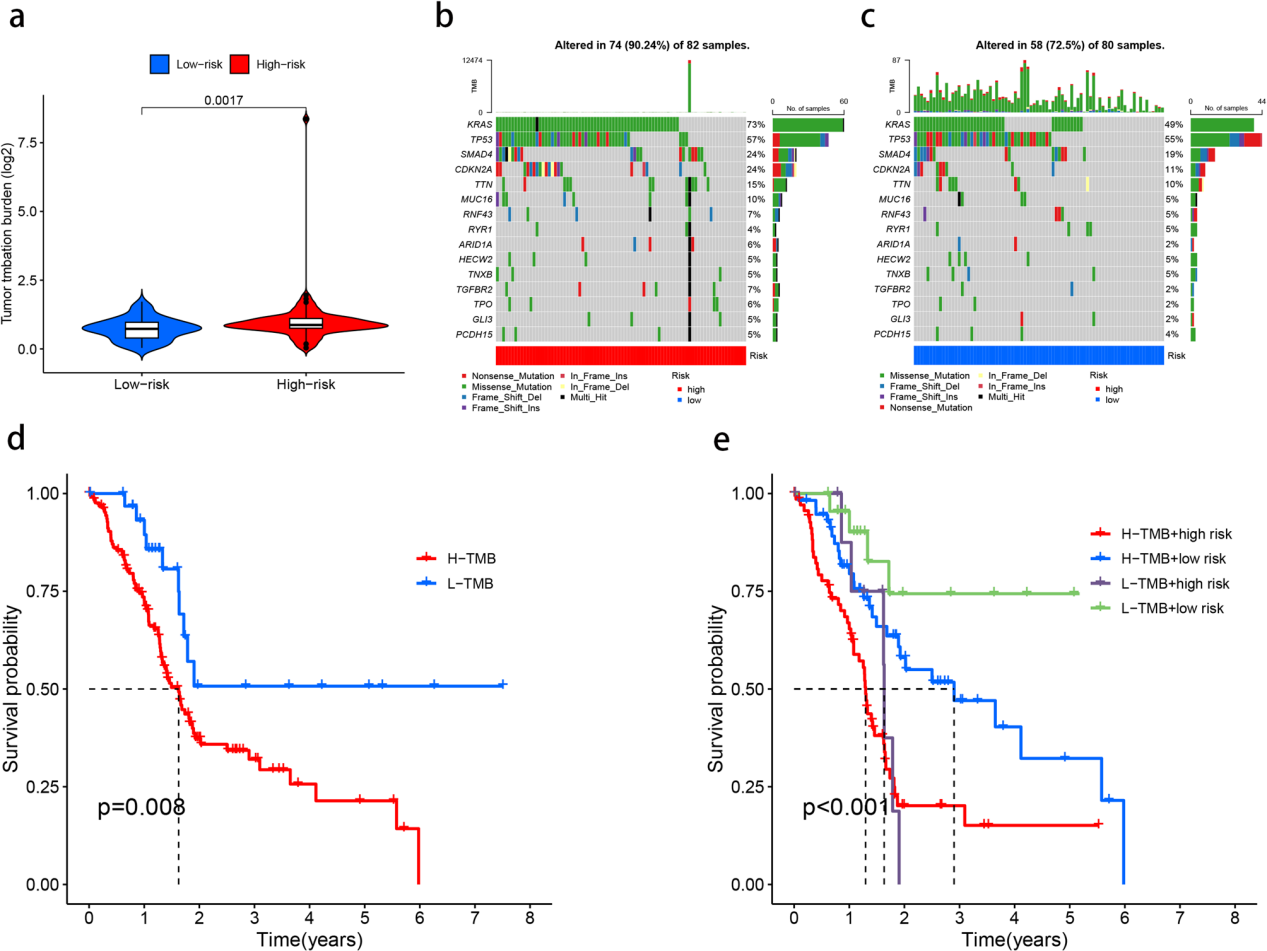
Tumor mutational burden (TMB) analysis. (**a**) TMB analysis of patients in high-risk group and low-risk group. (**b**,** c**) Mutational burden landscape of patients in high-risk group and low-risk group. (**d**) Kaplan–Meier survival curve shows that the OS of patients with low TMB (L-TMB) is longer than that of patients with high TMB (H-TMB). (**e**) Four groups of Kaplan–Meier survival analysis curves.

In the high and low risk subtypes, the mutation rates of several common mutated genes were calculated, and it was concluded that the high risk subtype had a higher mutation risk (Fig. 7a). The mutation frequencies of KRAS, TP53, SMAD4, and CDKN2A in high-risk group were 73%, 57%, 24% and 24%, respectively, while the mutation frequencies of KRAS, TP53, SMAD4 and CDKN2A in low-risk group were 49%, 55%, 19% and 11%, respectively (Fig. 7b and c). In order to further explore whether mutation has an impact on the survival of patients, we divided the samples into high and low mutation load groups according to the mutation load, and conducted survival analysis. The results showed that the survival rate of the high mutation load group was significantly lower than that of the low mutation load group (*P* = 0.008; Fig. 7d); The tumor mutation load was then combined with sample risk for survival analysis (*P* < 0.001; Fig. 7e), it is found that high mutation load with high risk means the lowest median survival time; Low mutation load with low risk means highest median survival time; High mutation with low risk showed a higher median survival time compared to low mutation with high risk.

### Drug sensitivity

**Fig. 8:**
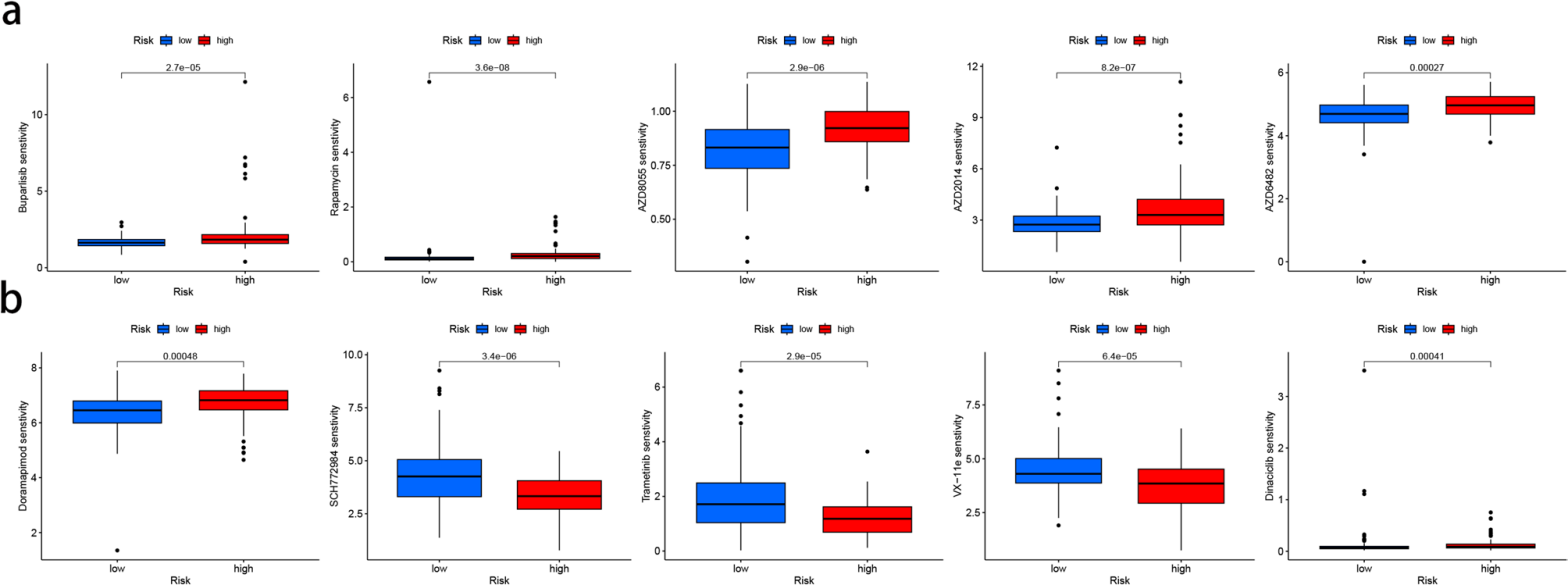
The sensitivity of pancreatic cancer to ten drugs. (**a**) The distribution of IC50 values shows a significant difference between patients in the low-group and high-risk group among Buparlisib, Rapamycinm, AZD8055, AZD2014, and AZD6482. (**b**) The distribution of IC50 values shows a significant difference between patients in the low-group and high-risk group among Doramapimod, SCH772984, Trametinib, VX-11e and CDK inhibitor Dinaciclib.

The description of the results of the oncoPredict analysis involved predicting drug sensitivity, and a lower IC50 value meant that the cells were more sensitive to the drug. Drug sensitivity analysis for high and low risk subtypes showed that pancreatic cancer had a lower IC50 value for PI3K/Akt/mTOR signal transduction pathway inhibitors Buparlisib, Rapamycinm, AZD8055, AZD2014, and AZD6482 (Fig. 8a). For Ras-Mitogen-Activated Protein Kinase (Ras-MAPK) Pathway inhibitors Doramapimod, SCH772984, Trametinib, VX-11e and CDK inhibitor Dinaciclib also showed low IC50 values (Fig. 8b). High-risk subtypes exhibit higher IC50 values for most drugs than low-risk subtypes, However, several drugs, such as CDK2/5/9 inhibitor Dinaciclib, MEK inhibitor Trametinib, ERK1/2 inhibitor SCH772984, ERK inhibitor VX-11e, showed lower IC50 value compared with low-risk subtypes.

### Differentially expressed genes (DEGs) and GO, KEGG, and GSEA functional enrichment analysis

**Fig. 9:**
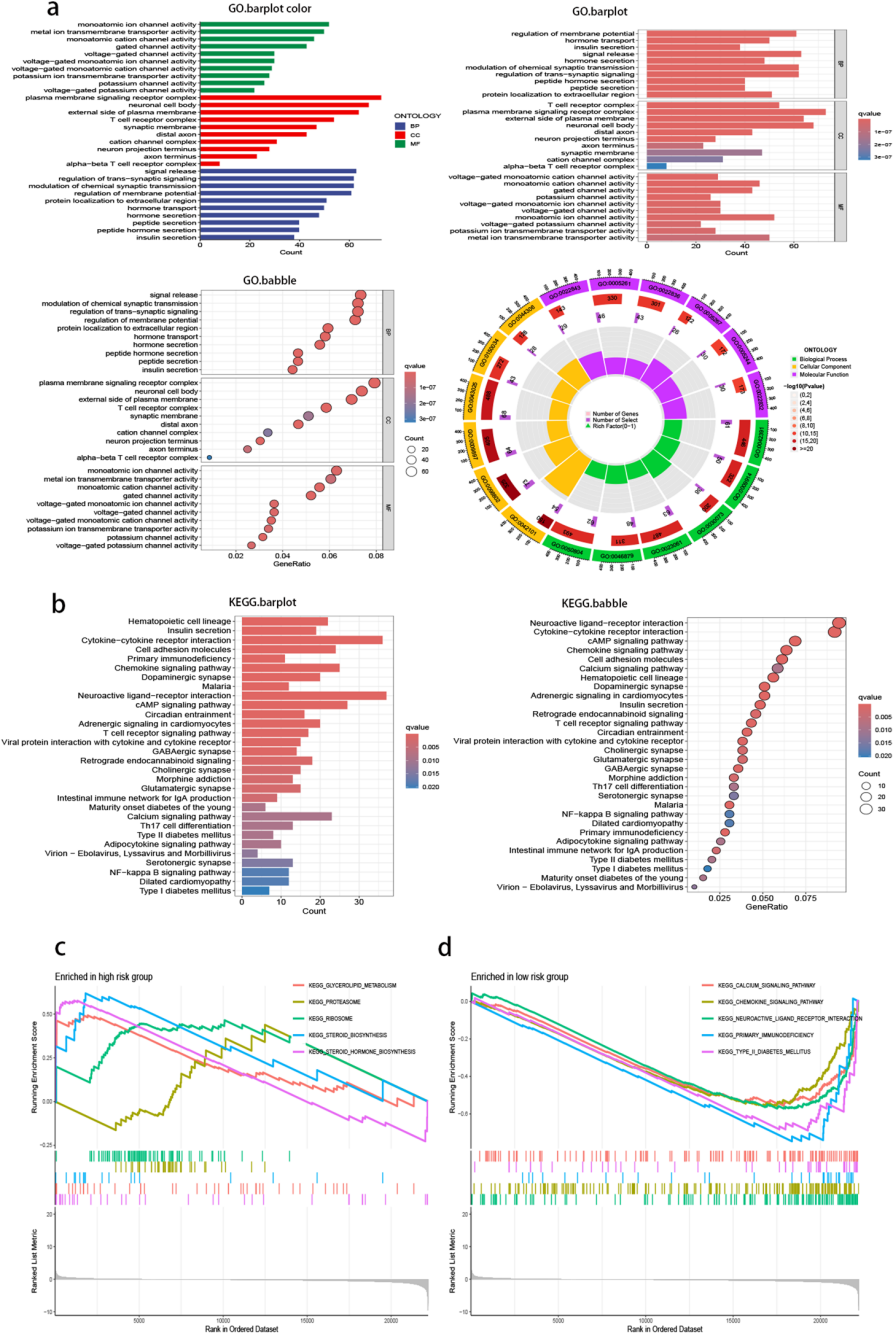
Functional enrichment analysis of differentially expressed genes (DEGs) in low-risk group and high-risk group. (**a**) GO enrichment analysis of DEGs. (**b**) The KEGG enrichment analysis. (**c**,** d**) Gene Set Enrichment Analysis.

DEGs between high- and low-risk subtypes underwent functional enrichment analysis. GO analysis indicated that these genes were primarily involved in ion channel activity, synaptic signaling, and membrane receptor complexes (Fig. 9a). KEGG pathway analysis highlighted enrichment in cytokine-cytokine receptor interaction and neuroactive ligand-receptor interaction (Fig. 9b)^16–18^. Notably, GSEA revealed that low-risk subtypes were significantly enriched in immune- and signaling-related pathways such as chemokine signaling pathway, calcium signaling pathway, and primary immunodeficiency. In contrast, high-risk subtypes were predominantly associated with metabolic processes, including glycerolipid metabolism and steroid hormone biosynthesis (Fig. 9c, d). These findings suggest distinct biological behaviors between the two subtypes, with the low-risk group exhibiting a more immunologically active phenotype.

### Clinical validation of MRLs prognostic risk model

**Fig. 10:**
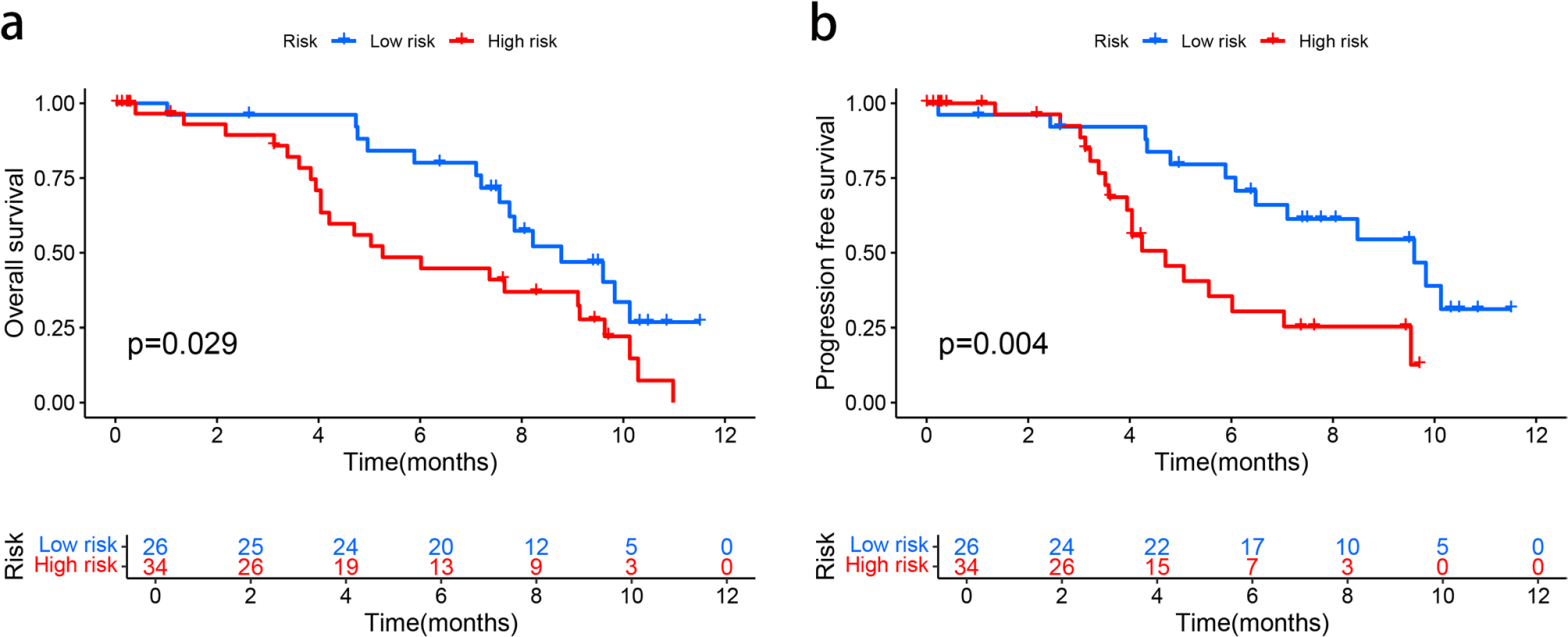
Clinical validation of MRLs Prognostic Risk Model. (**a**) Kaplan**–**Meier survival curve analysis shows the OS rates of low- and high-risk patients with PAAD(*P* = 0.029). (**b**) Kaplan**–**Meier survival curve analysis shows the PFS rates of low- and high-risk patients with PAAD(*P* = 0.004).

The tissues and clinical data of 60 pancreatic cancer patients were collected. The KM survival curve showed that there was a significant difference in PFS and OS between the low-risk group and the high-risk group (*p* < 0.05) (Fig. 10a and b).

## Discussion

In the last decade^19^, tumor-derived extracellular vesicles have become important mediators of intercellular communication between cancer cells and stromal cells in TME. Due to their involvement in various biological and pathological processes^20^, exosomes have attracted considerable attention in cancer research. Ma et al.^4^ defined migrasome as organelles in migrating cells, and Xu et al.^21,22^ established a comparison between exosomes and migrasome. The two structures differ in morphology and content, but both have been proposed to mediate intercellular communication^21^. However, until now, there has been relatively little research on the physiological role and cellular mechanisms of migrasome in cancer progression. This study is to investigate the role of migrasome in the prognosis of PAAD.

PAAD is essentially an immune cold tumor^23^ and all immunotherapies usually work by enhancing the anti-tumor effects of the existing immune system^24^. The lack of effective and reliable predictive biomarkers to monitor progression in PAAD has kept its high mortality and prognosis largely intact. Although the traditional anatomy-based TNM staging system promotes objective description and classification of the anatomical extent of malignant disease, making the TNM staging system and the degree of tumor differentiation widely used prognostic predictors. However, the conventional TNM staging system predicts the prognosis of PAAD patients with an accuracy of 0.60. Even with its continuous stepwise optimization, only minimal progress has been made^25^, which fails to meet the demand for accurate prediction. With the development of bioinformatics and the free sharing of databases, we try to find the characteristics and prognosis of pancreatic cancer from different dimensions. In this study, we found PAAD migrasome-related lncRNAs by co-expression analysis based on TCGA dataset and further constructed a prognostic model for risk characterization of four MRLs using LASSO Cox regression analysis. The model predicted the prognosis of patients from the perspective of migrasome. It was shown that the risk model based on migrasome-associated lncRNAs could significantly distinguish high- and low-risk patients and predict poor prognosis; meanwhile, the model could reflect the multidimensional biological characteristics, which could provide an important reference for tumor treatment^26^. Our results suggest that the four selected risk models for MRLs can be considered satisfactory independent prognostic indicators for predicting clinical outcomes in PAAD patients. Notably, the calibration curves of the 0.5-, 1-, and 3-year OS column chart models showed good agreement between column chart predictions and actual observations. In addition, the ROC curves for the pancreatic cancer migrasome lncRNA risk profile prognostic model had 1-, 3-, and 5-year AUC values above 0.65. And the Prognostic Risk Model were used in clinical to validate the accuracy. These results suggest that the identified pancreatic cancer MRLs risk model has a reliable predictive value for the prognosis of PAAD.

TME in PAAD hides complex interactions between tumor cells and stromal cells^27^. Numerous studies have reported^28^ that PAAD is highly infiltrated by immune cells. It has been demonstrated^22,29^ that macrophages can promote PAAD progression in vivo. And there is a close association between migrasome and PAAD macrophages. We observed more M0-type macrophage infiltration in TME with high risk subtypes of migrasomes. This may be due to the inducing effect of migrasomes on macrophages^22^. This was also confirmed by the study of Xu et al.^29^. Meanwhile, M0 macrophages are plastic and can polarize into pro-tumor M2 type or anti-tumor M1 type according to the cytokine microenvironment^30^. In the context of pancreatic cancer, tumor-associated macrophages (TAMs) often adopt an M2-like phenotype that promotes tumor progression, angiogenesis, and immunosuppression. The abundance of M0 macrophages in high-risk patients may represent a reservoir for M2 polarization, thereby fostering an immunosuppressive TME and facilitating tumor escape from immune surveillance. Conversely, low-risk patients demonstrated higher levels of naïve B cells and activated memory CD4 + T cells, which are indicative of a more active and potentially responsive immune environment^31,32^. Naïve B cells can contribute to antigen presentation and antibody production, while memory CD4 + T cells play a crucial role in sustaining cytotoxic CD8 + T cell responses and long-term immune memory. The enhanced activity of CD8 + T cells, helper T cells, and chemokine signaling in low-risk patients further supports a more robust anti-tumor immune response, which may explain their better survival outcomes. CD8 + T cells attack cancer cells by recognizing cancer antigen complexes bound by MHC class I molecules^33^. High CD8 + T-cell infiltration is considered a marker for “hot” tumors^34,35^. In the present study, CD8 + T cells in the low-risk subtype of TME had higher activity, and the low-risk subtype had a better prognosis. This is in agreement with Peltanova B et al.^36^. There are few studies on the relationship between migrasome and CD8 + T cells, and previous studies have shown^37^: extracellular vesicles inhibit the function of CD8 + T cells. Therefore, we hypothesized that in PAAD, migrasome would inhibit the function of CD8 + T cells, thus reducing anti-tumor immunity. These immune landscape differences also have implications for immunotherapy response. Although the high-risk group showed a higher TMB, which is generally associated with better response to immune checkpoint inhibitors, the predominance of immunosuppressive cells (e.g., M0 macrophages) may counteract this advantage. Conversely, the low-risk group, despite having a lower TMB, exhibited higher immune activity and checkpoint molecule expression, suggesting a potentially more favorable response to immune checkpoint inhibitors—a hypothesis supported by their lower TIDE scores, indicating less pronounced immune dysfunction and exclusion. In summary, we hypothesize that migrasome have an inducing effect on macrophages during the development of PAAD, and at the same time reduce the activity of CD8 + T cells in the tumor microenvironment, which leads to the further development as well as the deterioration of PAAD. Many immunosuppressive cells or molecules can mediate the immune escape of tumor cells by migrating to the tumor microenvironment^38^. Thus, the MRL-based risk model not only stratifies patients by prognosis but also reflects the underlying immune contexture of PAAD, offering insights into potential mechanisms of immune evasion and opportunities for tailored immunotherapeutic strategies.

Our functional enrichment analyses provide further mechanistic insights into the distinct immune phenotypes observed between the risk groups. The significant enrichment of pathways like “chemokine signaling” and “cytokine-cytokine receptor interaction” in the low-risk subgroup aligns perfectly with our findings of enhanced immune cell infiltration (e.g., CD8 + T cells, B cells) and antitumor immunity. Chemokines are fundamental for recruiting lymphocytes into the tumor bed^39^, which likely contributes to the more favorable “hot” tumor microenvironment and better prognosis in these patients. Conversely, the high-risk subgroup’s association with metabolic pathways such as “glycerolipid metabolism” and “steroid biosynthesis” is particularly intriguing. Reprogrammed metabolism is a recognized hallmark of cancer^40^, supporting rapid proliferation, survival, and immune evasion. Aberrant lipid metabolism, for instance, has been directly linked to immunosuppression in PAAD by altering the function of macrophages and T cells^41^. This metabolic reprogramming may be driven by, or synergize with, migrasome-mediated signaling. Although the direct connection between migrasomes and these specific metabolic pathways requires further validation, it is plausible that migrasomes released by aggressive tumor cells could carry oncogenic cargo (e.g., lipids, enzymes, or regulatory lncRNAs) that reprogram cellular metabolism in the TME, fostering a pro-tumorigenic and immunosuppressive state^42^. This integration of migrasome biology, metabolic reprogramming, and immune suppression offers a novel perspective on PAAD progression and may reveal potential therapeutic vulnerabilities for high-risk patients.

While the bioinformatics analyses presented in this study offer a comprehensive evaluation of the four-migrasome-related lncRNA (MRL) signature and its prognostic value in PAAD, we acknowledge several limitations that should be considered when interpreting our findings. Firstly, and most notably, the prognostic model constructed based on the four MRLs (MED14OS, AC141930.2, Z97832.2, LINC01091) lacks independent experimental validation using clinical samples. Although we have rigorously validated the model’s performance using internal bioinformatics approaches and a small external clinical cohort (as shown in Fig. 10), further verification of the expression levels of these lncRNAs through techniques such as qRT-PCR or in situ hybridization in a larger, independent cohort of PAAD tissues is essential to confirm their clinical utility and robustness. The limited availability of sufficient frozen PAAD tissue samples with complete long-term follow-up data at our current institution posed a significant challenge for such validation in this initial study. We explicitly acknowledge this as a primary limitation. Secondly, our study primarily reveals statistical associations between the MRL signature and the immune microenvironment, tumor mutational burden, and drug sensitivity. The specific biological functions and underlying molecular mechanisms through which these migrasome-related lncRNAs influence tumor progression and immune regulation in PAAD remain largely unexplored. To address these limitations and build upon our current work, we outline the following future research strategies: Prospective Sample Collection and Technical Validation: We are initiating a prospective, multi-center clinical study to systematically collect fresh-frozen PAAD tumor tissues and matched adjacent normal tissues, along with detailed clinical-pathological information and long-term outcome data. Using this well-annotated cohort, we will technically validate the expression patterns of the four MRLs using qRT-PCR and further explore their spatial localization within tumor tissues via RNA in situ hybridization (RNA-ISH). This will solidify the foundation for their clinical application. Functional Characterization In Vitro and In Vivo: To decipher the biological roles of these lncRNAs, we plan to conduct gain-of-function and loss-of-function experiments in PAAD cell lines. This will involve manipulating the expression of these MRLs (e.g., using siRNA/shRNA knockdown or plasmid overexpression) and assessing subsequent changes in cell migration, invasion, proliferation, and, importantly, their impact on the secretion and function of migrasomes. Furthermore, we will utilize xenograft mouse models to investigate how these MRLs influence tumor growth and metastasis in vivo, and to validate their effect on immune cell infiltration within the TME. Mechanistic Exploration: We will employ techniques such as RNA pulldown coupled with mass spectrometry, RNA immunoprecipitation (RIP), and chromatin isolation by RNA purification (ChIRP) to identify the potential protein-binding partners (e.g., transcription factors, chromatin modifiers) or downstream signaling pathways through which these lncRNAs exert their functions. Understanding whether they act as competing endogenous RNAs (ceRNAs) by sponging miRNAs will also be a focus. We believe that addressing these aspects in future studies will not only enhance the credibility of our prognostic model but also provide deeper insights into the functional significance of migrasome-related lncRNAs in PAAD pathogenesis, potentially uncovering novel therapeutic targets.

## Conclusion

In this study, we utilized data mining and bioinformatics methods to explore the immune landscape of pancreatic cancer. Our research established four migrasome-related lncRNA risk features, which can serve as independent prognostic indicators for PAAD patients, and demonstrated their important role in immune suppression within PAAD.

## Data Availability

The datasets analysed during the current study are available in the The Cancer Genome Atlas (TCGA) repository; (https://portal.gdc.cancer.gov/).

## Abbreviations

PAAD: Pancreatic adenocarcinoma
MRL: Migrasome-related LncRNA
ICI: Immune cell infiltration
ROC: Time-dependent receiver operating characteristic
AUC: Area under the curve
TME: Tumor microenvironment
HCC: Hepatocellular carcinoma
TCGA: The cancer genome atlas
OS: Overall survival
PFS: Progression-free survival
PCA: Principal component analysis
DEGs: Differentially expressed genes
TIDE: Tumor immune dysfunction and exclusion

## Declarations

### Approval for human experiments

This study was approved by International Traditional Medicine Clinical Trial Registry (ITMCTR, Registration No.: ITMCTR2025000430). All methods were carried out in accordance with relevant guidelines and regulations. The researcher confirming that informed consent was obtained from all subjects and/or their legal guardian(s).
